# Transcriptomic data sets examining several stress responses in *Zymomonas mobilis* strains

**DOI:** 10.1128/mra.01279-25

**Published:** 2026-02-25

**Authors:** Isabel Askenasy, Kevin S. Myers, Patricia J. Kiley

**Affiliations:** 1 DOE Great Lakes Bioenergy Research Center, University of Wisconsin-Madison 5228, Madison, Wisconsin, USA; 2 Department of Biomolecular Chemistry, University of Wisconsin-Madison 5228, Madison, Wisconsin, USA; 3 Wisconsin Energy Institute, University of Wisconsin-Madison, Madison, Wisconsin, USA; Indiana University Bloomington, Bloomington, Indiana, USA

**Keywords:** *Zymomonas mobilis*, RNA-seq, transcriptomics, stress response, bioenergy, anaerobic, aerobic

## Abstract

We used RNA-seq to compare gene expression from *Zymomonas mobilis* ZM4 grown under various conditions: aerobic ± paraquat, anaerobic ± hydrogen peroxide, or an iron chelator. We analyzed two mutant strains lacking predicted transcription factors (ZMO_0442 and ZMO_1411) grown under aerobic or anaerobic conditions. We report the RNA-seq data from these experiments.

## ANNOUNCEMENT

**Fig 1:**
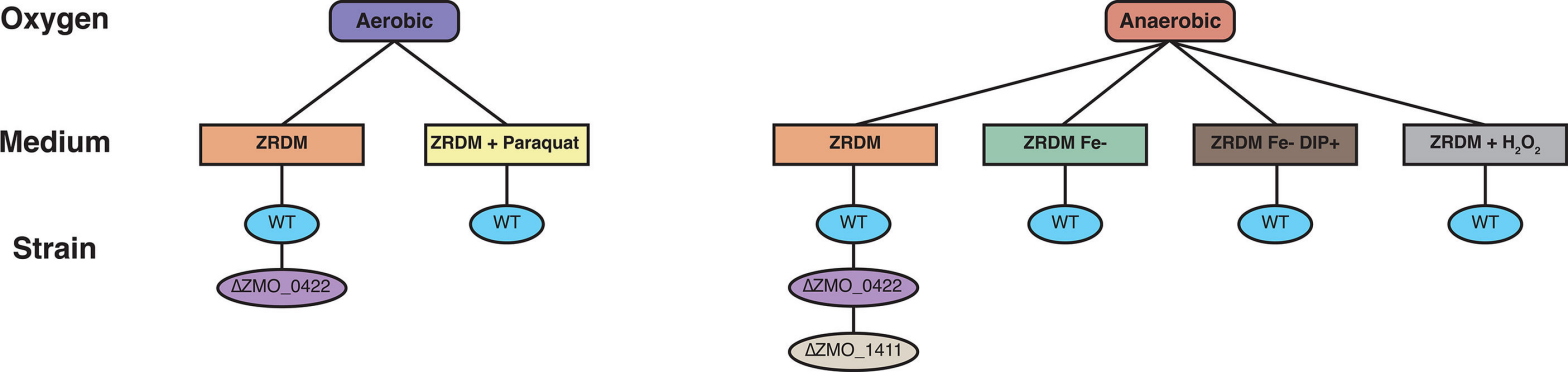
Growth conditions used for RNA-seq samples. A schematic shows the growth conditions for the RNA-seq samples described in terms of oxygen presence or absence (top line, rounded boxes), growth media (second line, boxes), and strains grown (bottom line, ovals). Full descriptions and replicate information can be found in Table 1.

*Zymomonas mobilis* ZM4 is an important, aero-tolerant gram-negative bacterium in bioenergy research due to its ability to produce and survive in large concentrations of ethanol (1), yet the transcriptional responses to many environmental stresses have not been extensively examined. We grew *Z. mobilis* under oxidative stress and iron limitation conditions and compared transcript abundance to non-stress conditions (Fig. 1). *Z. mobilis* RNA was obtained from WT and two mutant strains lacking putative stress response transcription factors: ∆ZMO_0422 (Rrf2 family) and ∆ZMO_1411 (Fur family). *Zymomonas* was grown in Rich Defined Medium (ZRDM) (2) buffered with 80 mM MES to pH 6.0 and supplemented with amino acids (5× supplement EZ, Teknova) and nucleobases (10× ACGU Solution, Teknova) at 30°C under aerobic (70% N_2_, 25% O_2_, and 5% CO_2_) and anaerobic (95% N_2_ and 5% CO_2_) conditions. To examine the response to low iron growth, anaerobic cultures were grown in ZRDM prepared without iron to mid-log phase when a subset was further treated with 5 µM α,α′-dipyridyl (DIP) for 20 min before collection. To examine the response to hydrogen peroxide, mid-log phase anaerobic cultures were treated with 15 µM hydrogen peroxide for 20 min before collection. To test the response to a redox cycling agent, anaerobic cultures at mid-log phase were shifted to aerobic growth for 20 min, and 1.5 mM paraquat was added for 20 min before collection. Ten milliliters of cells was added to 1 mL of cold 5% vol/vol phenol:ethanol mixture and centrifuged, and pellets were stored on dry ice. Nucleic acid was extracted using a hot phenol protocol and ethanol precipitation (3). DNA was removed by treatment with TURBO DNase (Thermo Fisher Scientific) at 37°C for 1 h. RNA was isolated using phenol extraction and ethanol precipitation or the Monarch Spin RNA Cleanup Kit following standard protocols (NEB) and resuspended in RNAse-free water. The concentration and quality were determined using a Qubit 2.0 Fluorometer (Thermo Fisher Scientific) and an Agilent TapeStation (Agilent Technologies), respectively.

Samples were submitted either to the Joint Genome Institute (JGI) or Azenta (formally GeneWiz) for library preparation and sequencing. Ribosomal RNA species were depleted using the QIAseq FastSelect bacteria kit (Qiagen). Strand-specific RNA sequencing libraries were prepared using the TruSeq stranded mRNA kit (Illumina) at JGI or the NEBNext Ultra II Directional RNA Library Prep Kit for Illumina (NEB) at Azenta. Briefly, first-strand synthesis was performed using random primers, and second-strand cDNA was synthesized using dUTP. cDNA fragments were adenylated at 3′ ends, and indexed adapters were ligated to cDNA fragments. Limited cycle PCR was used for library enrichment. The incorporated dUTP in the second-strand cDNA quenched the amplification of the second strand, preserving strand specificity. The sequencing library was validated on an Agilent TapeStation (Agilent Technologies) and quantified using a Qubit 2.0 Fluorometer (Thermo Fisher Scientific).

Samples were run using 2 × 150 bp paired-end configuration according to manufacturer’s instructions on an Illumina NovaSeq 6000 (JGI) or on an Illumina HiSeq 4000 (Azenta) (Table 1). Total reads from the samples ranged from 7,976,538 to 37,205,687 reads (Table 1). Raw FASTQ files without any additional filtering were uploaded to National Center for Biotechnology Information (NCBI) Sequence Read Archive (SRA). These data will expand our understanding of *Z. mobilis* stress responses and aid in engineering this important organism for various industrial applications.

**TABLE 1:** Summary of RNA-seq sample data^*a*^

Sample	Strain	Growth condition	Replicate number	Total paired reads	SRA accession number
ZM4-Dfur-1	*∆*ZMO_1411	Anaerobic	A	10,642,142	SRX29980582
ZM4-Dfur-2	*∆*ZMO_1411	Anaerobic	B	10,438,430	SRX29980583
ZM4-Dfur-3	*∆*ZMO_1411	Anaerobic	C	9,720,982	SRX29980584
ZM4-DrsuR-1	∆ZMO_0422	Anaerobic	A	9,469,602	SRX29980585
ZM4-DrsuR-2	∆ZMO_0422	Anaerobic	B	10,319,434	SRX29980586
ZM4-DrsuR-3	∆ZMO_0422	Anaerobic	C	11,521,078	SRX29980587
ZM4-DrsuR-4*	∆ZMO_0422	Anaerobic	D	37,305,687	SRX30019660
ZM4-DrsuR-5*	∆ZMO_0422	Anaerobic	E	34,391,133	SRX30019662
ZM4-DrsuR-6*	∆ZMO_0422	Anaerobic	F	31,539,542	SRX30019664
ZM4-DrsuR-1*	∆ZMO_0422	Aerobic	A	36,247,731	SRX30019659
ZM4-DrsuR-2*	∆ZMO_0422	Aerobic	B	37,305,687	SRX30019661
ZM4-DrsuR-3*	∆ZMO_0422	Aerobic	C	34,391,133	SRX30019663
ZM4-WT_PQ-1	WT	Aerobic + 1.5 mM Paraquat	A	10,500,872	SRX29980588
ZM4-WT_PQ-2	WT	Aerobic + 1.5 mM Paraquat	B	8,662,454	SRX29980589
ZM4-WT_PQ-3	WT	Aerobic + 1.5 mM Paraquat	C	12,165,538	SRX29980590
ZM4-WT_Control-NoFe-1	WT	Anaerobic + Fe-depleted media	A	10,276,196	SRX29980591
ZM4-WT_Control-NoFe-2	WT	Anaerobic + Fe-depleted media	B	12,580,238	SRX29980592
ZM4-WT_Control-NoFe-3	WT	Anaerobic + Fe-depleted media	C	10,570,882	SRX29980593
ZM4-WT_NoFe-DIP-1	WT	Anaerobic + Fe-depleted media + 5 µM DIP	A	7,940,926	SRX29980594
ZM4-WT_NoFe-DIP-2	WT	Anaerobic + Fe-depleted media + 5 µM DIP	B	10,610,018	SRX29980595
ZM4-WT_NoFe-DIP-3	WT	Anaerobic + Fe-depleted media + 5 µM DIP	C	14,345,688	SRX29980596
ZM4-WT_H2O2-1	WT	Anaerobic + 15 µM H_2_O_2_	A	11,526,450	SRX29980597
ZM4-WT_H2O2-2	WT	Anaerobic + 15 µM H_2_O_2_	B	8,050,962	SRX29980598
ZM4-WT_H2O2-3	WT	Anaerobic + 15 µM H_2_O_2_	C	10,209,482	SRX29980599
ZM4-WT_O2+control-1	WT	Aerobic	A	9,787,908	SRX29980600
ZM4-WT_O2+control-2	WT	Aerobic	B	8,658,572	SRX29980601
ZM4-WT_O2+control-3	WT	Aerobic	C	10,269,892	SRX29980602
ZM4-WT_O2+control-4*	WT	Aerobic	D	32,745,805	SRX30019665
ZM4-WT_O2+control-5*	WT	Aerobic	E	29,040,526	SRX30019670
ZM4-WT_O2+control-6*	WT	Aerobic	F	32,846,268	SRX30019668
ZM4-WT_O2-control-1	WT	Anaerobic	A	10,275,706	SRX29980603
ZM4-WT_O2-control-2	WT	Anaerobic	B	7,976,538	SRX29980604
ZM4-WT_O2-control-3	WT	Anaerobic	C	9,926,242	SRX29980605
ZM4-WT_O2-control-4*	WT	Anaerobic	D	30,272,223	SRX30019666
ZM4-WT_O2-control-5*	WT	Anaerobic	E	29,040,526	SRX30019669
ZM4-WT_O2-control-6*	WT	Anaerobic	F	32,846,268	SRX30019667

^*a*^ Sample names marked with * indicate samples sequenced at Azenta (formally GeneWiz). All other samples were sequenced at JGI.

## Data Availability

All RNA-seq files are available from NCBI GEO (accession numbers GSE304360 and GSE304389) and SRA (accession numbers PRJNA1300971 and PRJNA1301238).
